# Study on the expression of PAK4 and P54 protein in breast cancer

**DOI:** 10.1186/s12957-016-0913-6

**Published:** 2016-06-13

**Authors:** Yanqing Bi, Mengzi Tian, Jinghong Le, Linlin Wang, Xiaofang Liu, Jianhua Qu, Min Hao

**Affiliations:** Department of Breast and Thyroid Surgery, Dongying People’s Hospital, No. 317, Nanyi Road, Dongying, 257091 Shandong China; Department of General Surgery, Guangrao People’s Hospital, Dongying, Shandong China; Department of Pathology, Dongying People’s Hospital, Dongying, Shandong China

**Keywords:** PAK4, P54, Breast cancer

## Abstract

**Background:**

Previous evidence have demonstrated that p21-activated kinase PAK4 was correlated with breast cancer. The aim of this paper is to study the expression and interaction of p21-activated kinase (pAK)-4 and P54 protein in breast cancer.

**Methods:**

A total of 80 patients were enrolled in our study (breast fibroma *n* = 20, breast noninvasive cancer *n* = 20, early breast invasive cancer *n* = 20, and advanced breast invasive cancer). The expression of PAK4 was detected by immunohistochemical S-P method, and the relationship between them and the different pathological characteristics were compared. The subcellular localization of P54 and PAK4 in vitro was observed by immunofluorescence assay.

**Results:**

The expression of both PAK4 and P54 in breast cancer was much higher than that in breast fibroma. Meanwhile, we found that both PAK4 and P54 increased gradually as breast cancer progressed (advanced invasive > early invasive > noninvasive). The positive staining of P54 were mainly located in the cytoplasm, especially around the nucleus. There was no significant stained region in the cell matrix. The P54 localization in the cytoplasm was verified by confocal experiment, and the PAK4 was co-localized.

**Conclusions:**

PAK4 and P54 proteins may be used as molecular markers for diagnosis and treatment of breast cancer.

## Background

Breast cancer ranks the first among women malignant tumors, accounting for 7 to 10 % of the whole body cancer. Peripheral blood tumor marker detection is an important measure for early diagnosis of breast cancer. CA125, CEA, and TPS are the present common serum tumor markers. HER-2/Neu, estrogen receptor (ER), progesterone receptor (PR), epidermal growth factor receptor (EGFR), vascular endothelial growth factor receptor (VEGFR), and vascular endothelial growth factor (VEGF) are detected by pathological specimens. The positive rate and positive predictive value are not that satisfactory and are often used as reference index for clinical diagnosis and treatment. Along with advancement of scientific research, the pathways for phosphorylation/de-phosphorylation of proteins are found to be important for signal transduction, and their balance has a crucial role in the cell’s normal signal transduction and tumor occurrence or development. There have been some studies that p21-activated kinase (pAK)-4 has a high expression in breast cancer and ovarian cancer [1–3]. The purpose of our study was to explore the expression of PAK4 and P54 in breast cancer and the correlation of these proteins with pathological stages of breast cancer.

## Methods

### Clinical specimens

A total of 80 patients from April 2013 to April 2015 were enrolled in our study (breast fibroma *n* = 20, breast noninvasive cancer *n* = 20, early breast invasive cancer *n* = 20, and advanced breast invasive cancer).

The ages of patients with breast fibroma ranged from 30 to 72 years with a mean value of 44.5 ± 16.3 years. And the ages of patients with breast noninvasive cancer ranged from 29 to 66 years with a mean value of 42.5 ± 10.3 years. Among them, there were five ER-positive cases, four PR-positive cases, and three double-positive cases. The ages of patients with early breast invasive cancer ranged from 32 to 73 years with a mean value of 45.6 ± 14.2 years. Among them, there were four ER-positive cases, five PR-positive cases, and five double-positive cases. The ages of patients with advanced breast invasive cancer ranged from 46 to 77 years with a mean value of 52.3 ± 16.7 years. Among them, there were six ER-positive cases, four PR-positive cases, and four double-positive cases.

All the patients were treated for the first time without drug treatment, surgical resection, chemotherapy history, severe heart, liver, kidney, other organ dysfunction, or other malignant tumors, etc. For the study, we obtained the consent of the patients and their families, and this study was approved by the ethics committee of our hospital. For the same tissue, specimens were obtained from three different loci and completed by the same experienced expert, and the specimens were all fixed by 10 % formalin and embedded in paraffin with 4-μm continuous slicing.

### Immunohistochemistry S-P method

#### Main reagents and equipment

For this study, we used the following: rabbit IgG (Nanjing Kaiji Company) and peroxidase-labeled goat anti-rabbit IgG (Shenzhen Jinmei Biological Co., Ltd.), antibody dilutions (Wuhan Boshide Biotechnology Company), constitutive rafter acid salt buffer liquid powder (Nanjing Kaiji Company), and neutral gum (Shanghai Specimen Model Factory). Paraffin sections made from a tissue-slicing machine (Leica, Germany) were dewaxed in water; 3 % H_2_O_2_ was incubated at room temperature for 5 ~ 10 min, to eliminate endogenous peroxidase activity. The S-P immunohistochemical reagent kit was provided by Fujian Maixin Biotechnology Company.

### Procedure

The sections on the slides were rinsed three times in water and PBS, to keep the antigen intact (Shanghai Fengxian Shenxin Chemical Factory). After rinsing with PBS three times, goat serum (Guangzhou Jietewei Company) was flooded on sections for 20 min at room temperature. Subsequently, the first 50 μl of rabbit anti-human PAK4 polyclonal antibody (l: 200 dilution) (Germany BioGenes GmbH) was added to the sections and incubated at 37 °C for 1 ~ 2 h, followed by incubation at 4 °C overnight. The next day, the slides were washed with PBS at 37 °C for 45 min, rinsed three times with PBS. Next, biotin-labeled sheep anti-rabbit IgG antibody was added to sections at 37 °C and incubated for 10 to 30 min. It was followed by three times PBS washing, then HRP streptavidin solution was added and the slides were incubated at 37 °C for 10 to 30 min. The slides were rinsed with PBS three times, and DAB solution (Guangzhou Jietewei Company) was added for 5 ~ 10 min, followed by PBS wash for 10 min. Finally, the sections were counterstained with hematoxylin staining for 2 min, hydrochloric acid alcohol was differentiated, and ammonia turned back to blue. In the next step, the sections were washed with tap water for 10 ~ 15 min, dehydrated and transparent, and mounted for microscopic examination (OLYMPUS BX-4O optical microscope, Olympus, Japan).

### Scoring for stained sections

For interpretation of results, the Standard of Campo was followed (3). No color was given 0 points, weak color 1 point, moderate color 2 points, and strong color 3 points. Stained cells accounted for the total number of cells were less than 5 % for 0 points, 5 ~ 25 % for 1 point, 26 ~ 50 % for 2 points, and more than 51 % for 3 points. Finally, the results were determined by the total score of the percentage of positive cells and the staining intensity score: 0 points for negative, 1 to 2 points for weak positive, 3 to 4 points for medium positive, and more than 5 points for the strong positive. The results of the test were double blind-determined by two pathologists based on the same criteria. The different results were double blind-determined by another pathologist, and the mean value of the three persons was included in the statistics.

### Immunofluorescence

The cells were placed on a 12-hole cell culture plate, until the cells adhere to start expressing P54 and PAK4 protein plasmid; a double free DMEM was added to incubate for 4 h, which was later replaced with a DMEM culture medium, and the cells were cultured for 24 h, to guarantee outward plasmid normal protein expression. After 24 h, the culture medium was discarded and washed three times with PBS. The PBS was discarded, and the cells were fixed with 700 μl of 4 % paraform for 10 min. After three times washing with PBS, 0.1 % Triton-X-100 was added to permeabilize the cell at room temperature for 10 min. Next, again the slides were rinsed with PBS three times, followed by addition of 1 % sheep serum at room temperature for 1 h. Subsequently, the slides were incubated with the anti-PAK4 (1:2000) and anti-myc (1:2000) antibody at room temperature for 1 h and washed with PBS three times. Subsequently, Alexa-546 red mark goat anti-mouse antibody (1:5000) was added to incubate for l h in dark, followed by washing with PBS three times. Topro-3 nuclear staining was performed for 30 min and later washed with PBS three times. Finally, 50 % glycerol was added to the slides for mounting, to observe the subcellular localization of proteins in confocal laser scanning microscopy.

### Statistical analysis

SPSS20.0 software (SPSS Chicago, Ill) was used for the statistical analysis. All quantitative data were expressed as mean ± standard deviation. Comparison between groups was done using one-way ANOVA test followed by a post hoc test (LSD). Percentage (%) was used to express the enumeration data, and a chi-squared test was used for data analysis. The nonparametric total rank of independent samples of grade data was used to test. *p* values <0.05 were considered statistically significant

## Results

### Expression of PAK4 protein in different breast tissues

**Fig. 1:**
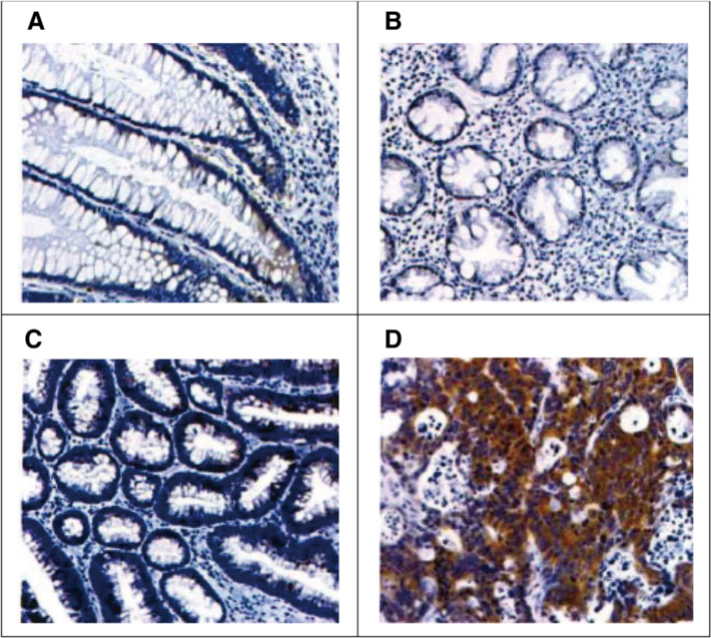
Expression of PAK4 protein in different breast tissues detected by immunohistochemistry. **a** Normal tissue. **b** Fibroma. **c** Metastatic tumor tissue. **d** Breast cancer

Expression of PAK4 in breast cancer was much higher than that in breast fibroma, and we found that PAK4 increased gradually as breast cancer progressed (advanced invasive > early invasive > noninvasive). The positive products were mainly located in the cytoplasm, especially obvious around the nucleus, and there was no significant dyeing in the cell matrix (*p* < 0.05) (Table 1, Fig. 1).

**Table 1:** Expression of PAK4 in different breast tissues

Group	No. of cases	Negative	Weakly positive	Moderately positive	Strong positive	Kruskal-Wallis	*p* value
Normal tissue	20	15	5	0	0	15.623	<0.001
Fibroma	20	10	7	3	0		
Metastatic tumor tissue	20	6	8	4	2		
Breast cancer	20	1	2	8	9

### Expression of PAK4 protein in different pathological types

Expression of P54 in breast cancer was much higher than that in breast fibroma, and we found that P54 increased gradually as breast cancer progressed (advanced invasive > early invasive > noninvasive). The differences were all statistically significant (*p* < 0.05) (Table 2).

**Table 2:** Expression of PAK4 in different pathological types

Pathological type	No. of cases	Negative	Weakly positive	Moderately positive	Strong positive	Kruskal-Wallis	*p* value
Noninvasive carcinoma	4	1	1	1	1	12.432	<0.001
Early invasive carcinoma	6	0	1	3	2		
Advanced invasive carcinoma	10	0	0	4	6		

### Cellular localization of P54 and PAK4 proteins

**Fig. 2:**
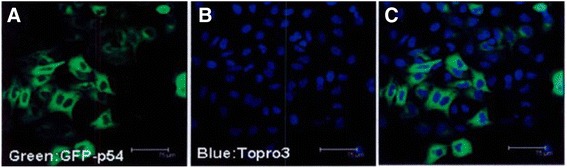
Subcellular localization of GFP-P54 protein. **a** *Green* GFP-p54. **b** *Blue* Topro3. **c** Merged

**Fig. 3:**
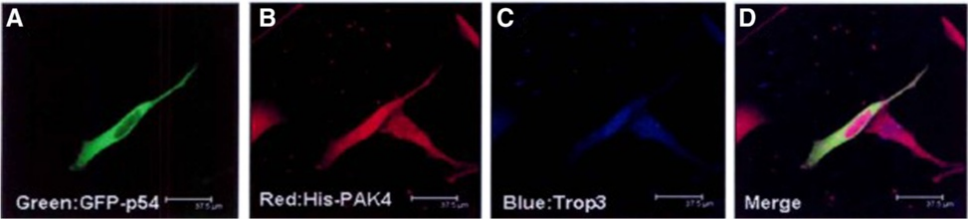
Cellular co-localization of GFP-P54 protein and PAK4 protein. **a** *Green* GFP-p54. **b** *Red* His-PAK4. **c** *Blue* Topro3. **d** Merged

P54 localization in the cytoplasm was verified by confocal experiment, and the PAK4 was co-localized as shown in Figs. 2 and 3. Expression of both PAK4 and P54 in breast cancer was much higher than that in breast fibroma and increased gradually as breast cancer progressed (advanced invasive 32.6 ± 8.2 % > early invasive 12.4 ± 3.5 % > noninvasive 2.0 ± 0.5 % > breast fibroma 0.2 ± 0.1 %; *F* = 20.325, *p* < 0.001).

## Discussion

PAK, P21 small GTP-activated kinase, is a serine/threonine protein kinase family. The molecular weight of 21kD, small GTP enzyme of Rho family, Cdc42, and Racl are the upstream molecules of PAKS, in the molecular structure; the end of N PAK is the regulation region of the kinase; the end of C is the substrate binding region with kinase activity [4]. PAK4 is a member of the PAK family, which is closely related to human tumor. It plays an important role in the repair of the cytoskeleton, the life of the normal cells, the positive regulation of some normal physiological metabolism, etc. [5–9]. In our study, expression of PAK4 in breast cancer was much higher than that in breast fibroma and increased gradually as breast cancer progressed (advanced invasive > early invasive > noninvasive). The positive products were mainly located in the cytoplasm, around the nucleus, as there was no significant staining in the cell matrix. The positive rate of PAK4 expression in noninvasive carcinoma, early invasive carcinoma, and advanced invasive carcinoma increased gradually, and the differences were all statistically significant. It shows that PAK4 may be used as a sensitive indicator and has important significance in judging the malignant degree and pathological types of breast cancer.

There is some research by constructing PAK4 eukaryotic expression vector and PAK4 ShRNA eukaryotic expression vector, transfecting breast cancer MDA-MB-231 cells, which showed that after up-regulation of PAK4 expression in breast cancer cells, it can significantly promote breast cancer cell proliferation; inhibit apoptosis; increase cell adhesion, movement, invasion, tumorigenesis, and other biological activity; and vice versa [10]. The normal mammary gland epithelium has active cell proliferation and cell apoptosis; in normal circumstances, there is a dynamic balance between cell proliferation and cell apoptosis, which is the main mechanism of breast mucosal removal of damaged cells [11, 12]. PAKs family plays an important role in the process of cell repair. Studies have shown that PAK4 is regulated by growth factors [13].

P54 protein is a kind of neuron-specific protein, and it is an effective microtubule instability factor and membrane-associated protein. P54 protein has a certain relationship with tumor, and P54 provides a new basis for the further improvement of PAK4 tumor signaling pathway [14, 15]. P54 as a member of the family has a structure of three functional regions: the membrane-binding region of the N end, the central regulatory region, and the coiled coil region of the C end. In the center of regulation of phosphorylation sites, there are phosphorylation sites of MAP and PKA kinase, and these phosphorylation sites can regulate microtubule depolymerization [16, 17].

Our study showed that the expression of P54 in breast cancer was much higher than that in breast fibroma and increased gradually as breast cancer progressed (advanced invasive > early invasive > noninvasive). We also found that P54 is located in the cytoplasm with PAK4 co-located. Two hybrid experiments have been shown through yeast, screen-interacting protein P54 and PAK4, and they were transferred to the AH109 yeast strain for yeast verification [18]. The results showed that both could interact; GST pull-down assay results further confirmed the interaction in vitro, and immune precipitation experiment verified the interaction in vivo. In summary, AK4 and P54 proteins may be used as molecular markers for diagnosis and treatment of breast cancer.

## Conclusions

PAK4 expression in breast cancer is adjacent normal tissues, breast fibroma, and breast cancer metastasis tissues, and breast cancer increased gradually. The positive staining was mainly located in the cytoplasm, especially more obvious around the nucleus. Besides, the positive rate of PAK4 expression in noninvasive carcinoma, early invasive carcinoma, and advanced invasive carcinoma also increased gradually. The P54 localization in the cytoplasm and the PAK4 was co-localized, so that PAK4 and P54 proteins may be used as molecular markers for diagnosis and treatment of breast cancer.
